# Diagnostics in Hymenoptera venom allergy: current concepts and developments with special focus on molecular allergy diagnostics

**DOI:** 10.1007/s40629-017-0014-2

**Published:** 2017-04-11

**Authors:** Thilo Jakob, David Rafei-Shamsabadi, Edzard Spillner, Sabine Müller

**Affiliations:** 1 Department of Dermatology and Venerology, Medical Center – University of Freiburg, Hauptstraße 7, 79104 Freiburg, Germany; 2 Immunological Engineering, Department of Engineering, Aarhus University, Aarhus, Denmark; 3 Department of Dermatology, Venerology and Allergology, University Hospital Gießen and Marburg, Gaffkystraße 14, 35392 Gießen, Germany

**Keywords:** Skin test, Recombinant allergens, cross-reactive carbohydrate determinants, Basophil activation test, Diagnostic algorithm

## Abstract

**Background:**

The high rate of asymptomatic sensitization to Hymenoptera venom, difficulty in correctly identifying Hymenoptera and loss of sensitization over time make an accurate diagnosis of Hymenoptera venom allergy challenging. Although routine diagnostic tests encompassing skin tests and the detection of venom-specific IgE antibodies with whole venom preparations are reliable, they offer insufficient precision in the case of double sensitized patients or in those with a history of sting anaphylaxis, in whom sensitization cannot be proven or only to the presumably wrong venom.

**Methods:**

Systematic literature research and review of current concepts of diagnostic testing in Hymenoptera venom allergy.

**Results and discussion:**

Improvements in diagnostic accuracy over recent years have mainly been due to the increasing use of molecular allergy diagnostics. Detection of specific IgE antibodies to marker and cross-reactive venom allergens improves the discrimination between genuine sensitization and cross-reactivity, and this provides a better rationale for prescribing venom immunotherapy. The basophil activation test has also increased diagnostic accuracy by reducing the number of Hymenoptera venom sensitizations overlooked with routine tests. This paper reviews current concepts of diagnostic testing in Hymenoptera venom allergy and suggests fields for further development.

## Introduction

Hymenoptera venom allergy (HVA) is one of the most common causes of anaphylaxis in adults and is frequently associated with severe anaphylaxis [1, 2]. It results in significant morbidity and impairment in quality of life [3]. A prevalence of up to 3.5% is reported in Europe [4]. Causal treatment in the form of venom immunotherapy (VIT) is effective and well tolerated.

In Germany the main perpetrators of HVA are yellow jackets (*Vespula*) and honeybees *(Apis*). Bumblebees (*Bombus*) and hornets (*Vespa*) are rarely involved in sting reactions and allergy is usually due to cross-reactivity to honeybee venom (HBV) and yellow jacket venom (YJV), respectively. In America and Mediterranean countries paper wasps (*Polistes*) or white-faced hornets (*Dolichovespula*) and some ants (*Formicidae*) are implicated in sting anaphylaxis but these are currently of little relevance in Germany.

According to the current guideline for diagnosis and therapy of bee and wasp venom allergy, only patients with a clinical history of anaphylactic sting reactions should undergo diagnostic testing, and only those with evidence of IgE-mediated sensitization to Hymenoptera venom should be offered VIT [5]. Making a confident diagnosis of HVA is complicated by several factors: The high rate of asymptomatic Hymenoptera venom sensitization in the general population, failure to identify or test for the culprit insect, loss of sensitization profiles over time and conditions mimicking anaphylaxis can all lead to an incorrect diagnosis. Up to 50% of those allergic to Hymenoptera venom are double sensitized to HBV and YJV but usually only one of these sensitizations is clinically relevant [6]. Often the insect responsible for the systemic reaction goes unidentified. When the insect was identified, it should be noted that the ability of the general population to correctly identify Hymenoptera is limited [7]. In order to minimize the risks of not detecting clinically relevant sensitizations, reliable diagnostic tests that accurately identify venom sensitizations are essential.

The diagnosis of HVA is based on a clinical history of Hymenoptera sting-related anaphylaxis and detection of IgE-mediated sensitization. Over recent years, the sensitivity of diagnostic tests has improved, largely due to increasing implementation of molecular allergy diagnostics and to some extent the use of the basophil activation test (BAT). Despite these improvements, current diagnostic tests are not without shortcomings. In particular, in the case of patients double sensitized to HBV and YJV and in those in whom no sensitization is detected, an accurate diagnosis of HVA remains challenging. In this paper we review the diagnostic tests currently available for the investigation of HVA, their benefits and limitations, and suggest areas for further improvement.

## Clinical history

Clinically irrelevant sensitizations to Hymenoptera venom occur in 27–40% of the general adult population and up to 50% of children [8–10]. It is important, therefore, to clarify if symptoms consistent with anaphylaxis occurred following a Hymenoptera sting. The risk of a systemic reaction in sensitized subjects with no previous history of HVA lies between 3.3 and 5% [10, 11]. Large local sting reactions occur in up to 26% of the general population and are defined as swellings of >10 cm in diameter lasting for >24 h [12]. In patients with previous large local reactions, the risk of a systemic reaction following a subsequent sting is reported to be less than 10% [13]. As this risk is low no diagnostic work-up is recommended. Similarly, unusual Hymenoptera sting reactions such as serum sickness like reactions or toxic reactions resulting from a large number of stings require no diagnostic work-up [5].

Symptoms of venom anaphylaxis usually occur within thirty minutes of the sting and are frequently associated with skin signs such as pruritus, flushing, urticarial, and angioedema [14]. Common gastrointestinal symptoms in Hymenoptera venom anaphylaxis are nausea and vomiting. Patients suffering anaphylaxis may report prodromal tingling of the palms and soles, restlessness, and a sense of impending doom. Severe anaphylaxis involves the respiratory and cardiovascular systems. Characteristic features are bronchoconstriction and dyspnea, tachycardia, hypotension, diaphoresis, and loss of consciousness. Urinary and fecal incontinence occur with profound circulatory dysregulation and the most severe systemic reactions result in cardiorespiratory arrest. When initial skin signs such as urticaria are followed by cardiovascular or respiratory symptoms, the clinical diagnosis of HVA is straightforward [14]. When this characteristic symptom evolution is absent, the diagnosis can be difficult. A number of conditions can simulate HVA, including chronic spontaneous urticaria, vasovagal syncope, anxiety disorders, cardiogenic shock, and arrhythmias. An incomplete list of differential diagnoses to be considered is shown in Table 1, adapted from [15]. In addition, the clinician should be alert to anaphylaxis featuring predominant circulatory dysregulation without skin signs. This pattern is often observed in patients with underlying clonal mast cell disorders that may have a normal baseline serum tryptase level [16]. The Spanish Network on Mastocytosis (Red Española de Mastocitosis) has developed a scoring system that may help to identify such patients [17, 18].

**Table 1:** Conditions that can cause symptoms mimicking anaphylaxis, modified from the guideline for acute therapy and management of anaphylaxis, adapted from [15]

Cardiac arrhythmias
Hypertensive crisis
Pulmonary embolism
Status asthmaticus
Tracheobronchial obstruction
Carcinoid syndrome
Pheochromocytoma
Hypoglycemia
Dissociative disorders and conversion (e. g., Globus hystericus)
Somatoform disorders (e. g., psychogenic dyspnea, “vocal cord dysfunction”)
Seizure disorders
Hereditary/acquired angioedema
Intoxication (alcohol, opioids, histaminosis)

The identity of culprit insect should be clarified. A recent study assessing the accuracy of the general population in identifying four different Hymenoptera species showed almost one third failed to correctly identify yellow jackets, half failed to identify *Polistes* and approximately 10% did not recognize honeybees [7]. Therefore, it is important to remain skeptical regarding the patients’ account of the culprit insect. It is often assumed the culprit insect can be identified based on the whether or not the stinger remains in the skin following injection. Due to structural differences, the sting apparatus of a honeybee is more likely than that of a yellow jacket to lodge in the skin. However, whether or not a stinger remains in the skin is influenced by skin characteristics at the sting site. Information on the remaining of a stinger is indicative but not reliable for identifying the stinging insect.

## Skin testing

In some countries skin testing is considered the gold standard [19, 20]. In Europe standardized, dialyzed whole venom preparations are available for honey- and bumblebee, yellow jacket, hornet, *Polistes*, and *Dolichovespula*. The process of dialysis removes low molecular weight substances such as biogenic amines that cause nonspecific test reactions. In Germany, bumblebees, hornets, *Polistes*, and *Dolichovespula* are rarely the primary sensitizer in HVA. It is usually sufficient to test with HBV and YJV preparations. Immigrants from Mediterranean or American countries, however, may be primarily sensitized to *Polistes* and/or *Dolichovespula*. In this case testing with further venoms should be considered.

The skin prick test (SPT) is quick, simple to perform, and inexpensive. As severe systemic reactions have occurred following intradermal tests, it has been recommended that intradermal tests should be preceded by a SPT. In 2013 the safety and efficacy of simultaneous intradermal testing in 478 Hymenoptera venom allergic patients with 0.02 ml of 0.001, 0.01, 0.1, and 1.0 μg/ml of HBV and YJV was assessed. A systemic reaction incidence of 0.6% was reported [21] but no severe reactions occurred and none of the reactions could have been prevented by stepwise testing. A recent study of 300 patients with suspected HVA in which skin testing consisted of simultaneous intradermal tests with 0.02 ml of 1.0 µg/ml of five different commercially available venom preparations reported one delayed adverse reaction [22]. Several different protocols for skin testing with Hymenoptera venom exist. Currently used approaches are summarized in Table 2 [5, 21]. Despite the low risk of systemic reactions, the current German guideline for diagnosis and therapy of bee and wasp venom allergy recommends stepwise skin testing in patients with a history of severe anaphylaxis [5].

**Table 2:** Skin tests with HBV and YJV may be carried out in a stepwise manner or simultaneously depending on severity of anaphylaxis and individual patient risk factors [5, 21]

1)	Skin prick test: 1, 10, 100 µg/ml and intradermal test 1 µg/ml
2)	Skin prick test: 1, 10, 100, 300 µg/ml
3)	Intradermal tests: 0.001; 0.01; 0.1; and 1 µg/ml

When interpreting skin tests it is important to know the temporal relationship to the anaphylactic sting event. Skin testing directly after the event should be avoided, since tachyphylaxis may result in false negative results. Most reliable results are obtained 1–6 weeks after the sting event, most likely due to boostering of the relevant venom-specific IgE antibodies through the sting. The rate of loss of sensitization to Hymenoptera venom in skin tests is reported to be 12% per year, with 33% of skin tests becoming negative after 2.5 years [23]. While sensitization remains detectable for many years in a number of patients, negative results may merely reflect a long latency between sting event and diagnostic testing. The use of medications such as corticosteroids, antihistamines, and antihistaminergic antipsychotics can suppress skin test responsiveness and give rise to false negative results. Table 3 lists medications that should be discontinued prior to skin testing, adapted from reviews [24, 25].

**Table 3:** Frequently used medicaments that suppress skin tests together with the duration for which they should be discontinued prior to testing are listed, adapted from [24, 25]

Drug group	Suppression	Period of discontinuation
*H1 Antihistamine 1* ^st^ *generation*	+++	>3 days
*H1 Antihistamine new generation*	+++	>7 days
*H2 Antihistamine*	−/+	2 days
*Ketotifen (mast cell stabilizer)*	+++	>5 days
*Topical glucocorticosteroid (GCS) in the test area >4 weeks*	+	>1 week
*Systemic short-term GCS*		
<50 mg prednisolone	−	3 days
>50 mg prednisolone	−/(+)	>1 week
*Systemic long-term GCS*		
<10 mg prednisolone	−	0
>10 mg prednisolone	−/+	>3 weeks
*Benzodiazepines*	+++	>7 days
*Omalizumab*	+++	4–8 weeks
*Tricyclic antidepressants*	+++	>14 days
*Promethazine (neuroleptic)*	++	>5 days

## Specific IgE antibodies to whole venom preparations

Detecting specific IgE antibodies (sIgE) to whole insect venoms is one of the main diagnostic methods in HVA. At the same time, detection of sIgE to insect venoms is an analytical measurement allowing only the presence or absence of IgE-mediated sensitization to be detected. A diagnosis of HVA can only be made in conjunction with the patient’s clinical history.

For the detection of specific IgE to whole venom, various test systems encompassing liquid or solid phase systems and single or multiplex tests are available. The sensitivity of sIgE to HBV in HBV allergic patients is reported to be high (98–100%) [26, 27]. The reported sensitivity of sIgE to YJV is lower (83–93%) [26–28]. It is of limited use to calculate diagnostic specificity and positive predictive values for detecting HVA when evaluating the performance of sIgE testing, since the test system only enables the presence or absence of IgE-mediated sensitizations to be detected and cannot assess clinical relevance.

As with skin tests, in order to make use of the booster effect, venom sIgE should be measured 1–6 weeks after a sting event [5, 12, 29]. Failure to detect venom sIgE in patients with a convincing history of Hymenoptera venom anaphylaxis may be due to a long time interval between the sting reaction and diagnostic work-up. sIgE has been found to decrease between 1 and 4 years after Hymenoptera venom anaphylaxis and may fall below the level of detection with very long latency periods. Earlier assumptions that venom sIgE is consumed by an anaphylactic sting reaction have not been verified [12]. Boostering of venom sIgE following stings from Hymenoptera, to which the patient is not allergic, may give rise to false-positive results.

The internationally accepted cut-off level for detecting sIgE is 0.35 kU/l, however, the analytical sensitivity of modern assays is 0.10 kU/l [30]. As the level of venom sIgE is related to total IgE, venom sIgE between 0.10 and 0.35 kU/l may be clinically relevant in patients with low total IgE and this must be evaluated in the context of the patient history.

## An introduction to Hymenoptera venom allergens

**Fig. 1:**
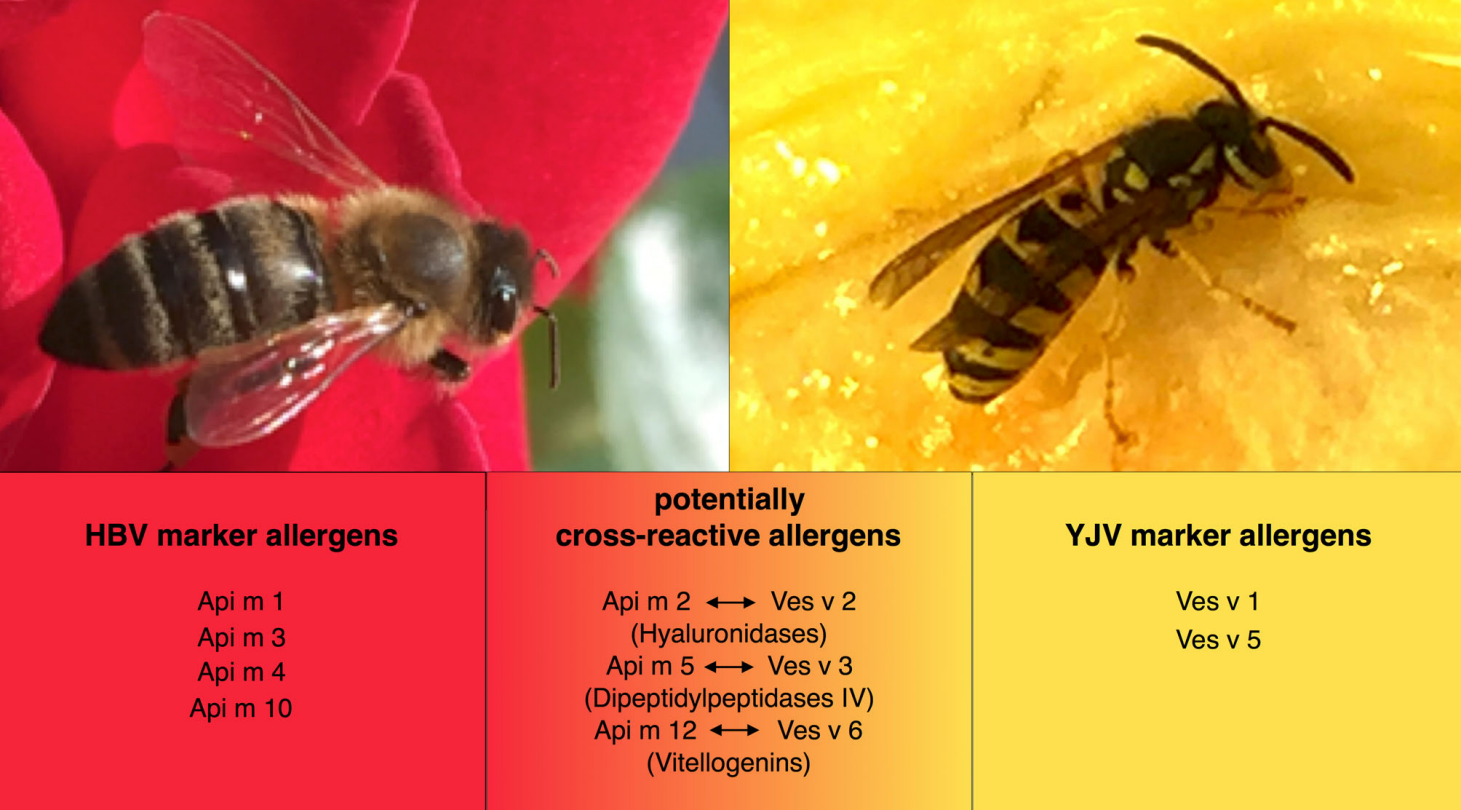
Honeybee and yellow jacket venom and their respective marker and cross-reactive allergens. *Apis mellifera* marker allergens: *Api m 1, 3, 4* and *10*; *Apis mellifera* potentially cross-reactive allergens: *Api m 2, 5* and *12*. *Vespula vulgaris* marker allergens: *Ves v 1* and *5*; *Vespula vulgaris* potentially cross-reactive allergens: *Ves v 2, 3* and *6*. *HVB* honeybee venom, *YJV* yellow jacket venom, *Api m 1* *Apis mellifera* allergen number 1, *Ves v 1* *Vespula vulgaris* allergen number 1

Currently 12 honeybee and 5 yellow jacket venom allergens have been characterized in detail and are listed in the official allergen data bank of WHO/IUIS subcommittee on allergen nomenclature [31]. Some allergens present in HBV are specific to honeybee and are not present in the venom of yellow jacket or other Hymenoptera. These are termed marker allergens as they serve as a marker of genuine sensitization to HBV. Examples of honeybee marker allergens are phospholipase A2 (Api m 1), acid phosphatase (Api m 3), melittin (Api m 4), and icarapin (Api m 10). In YJV, phospholipase A1 (Ves v 1) and antigen 5 (Ves v 5) are marker allergens specific to yellow jacket. In addition, some allergens in HBV are similar to allergens in YJV resulting from a high sequence identity. Such allergens are termed homologous or cross-reactive allergens as sIgE of individuals sensitized to one of these allergens in HBV might show cross-reactivity with the homologous allergens in YJV. Cross-reactive allergens in HBV and YJV are the hyaluronidases (Api m 2 and Ves v 2), the dipeptidylpeptidases IV (Api m 5 and Ves v 3), and the vitellogenins (Api m 12 and Ves v 6). Fig. 1 illustrates marker and cross-reactive allergens present in HBV and YJV, respectively. The majority of HBV and YJV allergens are glycoproteins containing one or more oligosaccharides linked to the protein. These carbohydrates often contain an alpha 1.3-linked fucose residue on the N‑glycan core that is produced by insects and plants. The resulting structure is known as cross-reactive carbohydrate determinant (CCD) and does not exist in mammals. As a result and due to their widespread prevalence, CCD are highly immunogenic epitopes that can give rise to the production of sIgE. The clinical relevance of CCD is disputed but the consensus in the case of HVA is that CCD are clinically irrelevant. Table 4 shows an overview of Hymenoptera venom allergens relevant in Germany and Europe including the number of potential glycosylations sites (adapted from [32]).

**Table 4:** Overview of Hymenoptera venom allergens relevant in Europe (adapted from [32])

Allergen	Name/function	MW (kDa)	Potential N‑glycosylations
*Honeybee allergens (Apis spp.)*			
Api m 1	Phospholipase A2	17	1
Api m 2^a^	Hyaluronidase	45	3
Api m 3	Acid phosphatase	49	2
Api m 4	Melittin	3	–
Api m 5^b^	Allergen C/DPP IV	100	6
Api m 6	Protease inhibitor	8	–
Api m 7^c^	Protease	39	3
Api m 8	Carboxylesterase	70	4
Api m 9	Carboxypeptidase	60	4
Api m 10	CRP/Icarapin	55	2
Api m 11.0101	MRJP 8	65	6
Api m 11.0201	MRJP 9	60	3
Api m 12^d^	Vitellogenin	200	1
*Bumblebee allergens (Bombus spp.)*			
Bom p 1, Bom t 1	Phospholipase A2	16	1
Bom p 4, Bom t 4	Protease	27	0.1
*Yellow jacket allergens (Vespula spp.)*			
Ves v 1	Phospholipase A1	35	–
Ves v 2.0101^a^	Hyaluronidase	45	4
Ves v 2.0201^a^	Hyaluronidase	45	2
Ves v 3^b^	DPP IV	100	6
Ves v 5	Antigen 5	25	–
Ves v 6^ d^	Vitellogenin	200	4
*Bald-faced hornet allergens (Dolichovespula spp.)*			
Dol m 1	Phospholipase A1	34	2
Dol m 2	Hyaluronidase	42	2
Dol m 5	Antigen 5	23	0
*Hornet allergens (Vespa spp.)*			
Vesp c 1	Phospholipase A1	34	0
Vesp ma 2	Hyaluronidase	35	4
Vesp c 5	Antigen 5	23	0
*European wasp allergens (Polistes spp.)*			
Pol d 1	Phospholipase A1	34	1
Pol d 4	Protease	33	6
Pol d 5	Antigen 5	23	0

*MW* molecular weight

^a,b,d^Refer to homologous allergens

^c^A homologous yellow jacket protease and further honeybee proteases were identified but these have not been described as allergens

Between 45 and 50% of Hymenoptera venom allergic patients display double sensitization to both HBV and YJV on diagnostic testing with skin tests and venom sIgE. This makes choosing the correct venom for immunotherapy difficult, when the culprit insect is unknown [6]. Double sensitization to HBV and YJV occurs for three reasons and molecular allergy diagnostics can help clarify the relevance of sensitizations by measuring sIgE to individual allergens present in whole HBV and YJV.

First, double sensitization can represent a genuine double sensitization to both HBV and YJV marker allergens, resulting from previous stings from both of these insects. The clinical relevance of genuine double sensitization depends on the patient history. Second, double sensitization can result from IgE-mediated sensitization to cross-reactive, homologous venom allergens present in HBV and YJV, resulting from a sting from either of the insects. The third and most common cause of double sensitization to HBV and YJV is the presence of sIgE to CCD. This accounts for up to 50% of double sensitizations to HBV and YJV [33]. Specific IgE to CCD can be measured using horseradish peroxidase (HRP) or the glycan structure from pineapple stem bromelain (MUXF3) as test allergen and should be included in the investigation of double sensitized patients. However, sensitization to CCD does not rule out a simultaneous clinically relevant sensitization to an allergen protein epitope [32, 34].

## Molecular allergy diagnostics in HVA

Recombinant expression of allergens has enabled the production of CCD-free allergens for diagnostic purposes [35]. As a result, molecular allergy diagnostics have become an integral part of HVA diagnostics. Currently, a limited number of recombinant allergens are commercially available: rVes v 1 and rVes v 5 in the case of YJV and rApi m 1, rApi m 2 and recently rApi m 10 in the case of HBV.

With currently available test systems, sensitization rates of between 85 and 90% for rVes v 5 [28, 36–40] and between 39 and 79% for rVes v 1 are reported [35, 36, 39, 40]. Combining both allergens resulted in a sensitivity of 92–96% for the detection of YJV allergic patients [28, 35, 36, 38–40]. It was previously shown that yellow jacket allergic patients not sensitized to whole YJV subsequently tested positive for rVes v 5 [37, 41]. An increased diagnostic sensitivity of 97% was reported for the detection of sIgE to a rVes v 5 supplemented whole YJV extract compared to 83% using conventional whole YJV [28]. These results led to the spiking of YJV with rVes v 5 by one manufacturer and since October 2012 this spiked YJV preparation has fully replaced that previously commercially available. The remaining currently identified YJV allergens show potential cross-reactivity with homologous allergens in HBV. Studies assessing improved diagnostic precision by detecting sIgE to cross-reactive allergens gave mixed results. Diagnostic testing with ImmunoCAPs and ELISA for the detection of sensitizations to rVes v 1, 2, 3, and 5 allowed a YJV sensitization to be found in 84% of YJV allergic patients who had tested negative using whole YJV extract (*n* = 19). In HBV allergic patients serologically nonreactive to whole HBV extract, the same study detected sensitizations to rApi m 1, 2, 3, and 5 in 100% (*n* = 8) [36], suggesting that testing with single components may be more sensitive to detect IgE sensitizations in Hymenoptera venom allergy. This assumption, however, could not be confirmed in a follow-up study with a higher number of patients tested at our center [42]. Currently no marker allergens specific to *Polistes* or *Dolichovespula* have been identified so that patients primarily sensitized to these Hymenoptera venoms will easily be misdiagnosed as allergic to yellow jacket but subsequently inadequately protected by yellow jacket VIT [43].

Phospholipase A 2 (Api m 1) was the first marker allergen to be identified in HBV. Compared to Ves v 5 in the case of YJV allergic patients the sensitivity of Api m 1 in HBV allergy is low. In HBV allergic patients, the prevalence of sensitization to Api m 1 is reported to range between 57 and 97% [26, 37, 44–47]. Based on this, lack of sensitization to Api m 1 in patients suspected of having HBV allergy is insufficient to rule out genuine HBV sensitization. The reported difference in Api m 1 sensitization rates may reflect regional differences as suggested by some [48] or may reflect differences in the definition of the patient population as suggested by others [37, 40]. In addition, the sensitivity of Api m 1 may partly depend on the test system used. Recently, direct comparison of sIgE levels to Api m 1 measured on the Immulite fluid phase test system and the ImmunoCAP solid phase test system suggested a higher sensitivity for the Immulite system [49, 50]. It was speculated that IgE binding capacity of the recombinant Api m 1 used in the ImmunoCAP system may be diminished due to altered protein folding [49, 50]. However, this seems rather unlikely, since direct comparison of IgE reactivity to natural Api m 1 and to the recombinant Api m 1 on the ImmunoCAP system has been shown to be identical in CCD-negative sera [51]. Another suggested cause is possible variance in the interpolation calibration algorithm between the assays [49]. Indeed, two comparative studies using chimeric mouse human IgE antibodies to a variety of different recombinant allergens have provided convincing evidence that the Immulite system tends to overestimate the actual levels of sIgE to a given allergen approximately 3–5 fold [52, 53]. Thus, as concluded by one of the studies [52], just because two systems present their results in the same units does not mean that the results are necessarily correct or interchangeable.

Further allergens occurring in lesser abundance in HBV have since been identified as major allergens including Api m 3 and Api m 10. Sensitizations to these allergens are present in 50 and 62% of HBV allergic patients, respectively. An extended repertoire of HBV marker allergens (Api m 1, Api m 3, Api m 4, and Api m 10) significantly increased the diagnostic sensitivity for detection of HBV sensitization and reached nearly 90% compared to 72% for Api m 1 alone [46]. In addition, a high individual heterogeneity of sensitization profiles to HBV allergens was found. Similarly in patients double sensitized to HBV and YJV that had not identified the culprit insect, the combination of Api m 1, Api m 3, and Api m 10 increased the diagnostic sensitivity to 78.6% compared with 54% using Api m 1 alone. Sensitizations to Api m 3 and Api m 10 were detected in two thirds of patients that had tested negative to Api m 1, thus, providing evidence of the need for treatment with both honeybee and yellow jacket VIT in these patients [54]. In Table 5 reported sensitization rates to HBV and YJV allergens and combinations of allergens are shown.

**Table 5:** Depicts sensitization rates to honeybee and yellow jacket venom allergens in Hymenoptera venom allergic patients as reported in the literature

Allergen source/allergens	Name/function	Sensitization frequency (%)	No. of patients	Reference
*Apis mellifera*				
rApi m 1	Phospholipase A2	79 57 78 78 72 97	34 175 100 23 144 100	Hofmann 2011 [37] Korosec 2011 [45] Sturm 2011 [47] Muller 2012 [44] Kohler 2014 [46] Muller 2009 [26]
rApi m 2	Hyaluronidase	46 52 48	82 40 144	Hofmann 2011 [37] Sturm 2011 [47] Kohler 2014 [46]
rApi m 3	Acid phosphatase	38 50	40 144	Grunwald 2006 [55] Kohler 2014 [46]
nApi m 4	Melittin	27 42 23	82 40 144	Hofmann 2011 [37] Sturm 2011 [47] Kohler 2014 [46]
rApi m 5	Dipeptidylpeptidase IV	60 58	35 144	Blank 2010 [56] Kohler 2014 [46]
rApi m 6	Serine protease inhibitor	26	31	McIntyre 2012 [57]
rApi m 10	Icarapin	49 62	68 144	Blank 2011 [58] Kohler 2014 [46]
rApi m 11a (0101) rApi m 11b (0201)	Major royal jelly protein 8/9	15/34	47	Blank 2012 [59]
rApi m 12	Vitellogenin	44	45	Blank 2013 [60]
Combination	rApi m 1, rApi m 2, rApi m 3, nApi m 4, rApi m 5, rApim 10	94	144	Kohler 2014 [46]
Combination	rApi m 1, rApi m 2, rApi m 3, rApi m 5	92	86	Cifuentes 2014 [36]
*Vespula vulgaris*				
rVes v 1	Phospholipase A1	79 54 39 58	14 148 86 109	Seismann 2010 [35] Ebo 2013 [39] Cifuentes 2014 [36] Hofmann 2011 [40]
rVes v 2a (0101) rVes v 2b (0201)	Hyaluronidase Hyaluronidase**inactive isoform	5 28 20	41 86 41	Seismann 2010 [35] Cifuentes 2014 [36] Seismann 2010 [35]
rVes v 3	Dipeptidylpeptidase IV	57 50	35 86	Blank 2010 [56] Cifuentes 2014 [36]
rVes v 5	Antigen 5	90 90 90 87 85 90	59 148 308 86 200 109	Hofmann 2011 [37] Ebo 2013 [39] Vos 2013 [28] Cifuentes 2014 [36] Korosec 2012 [38] Hofmann 2011 [40]
rVes v 6	Vitellogenin	39	28	Blank 2013 [59]
Combination	rVes v 1 + r Ves v 5	93 92 98 96 96	14 200 148 308 109	Seismann 2010 [35] Korosec 2012 [38] Ebo 2013 [39] Vos 2013 [28] Hofmann 2011 [40]
Combination	rVes v 1, rVes v 2, Ves v 3, rVes v 5	95	86	Cifuentes 2014 [36]

From our own data, up to 10% of patients with a convincing history of HVA will have negative skin tests. In some of these, venom sIgE will also be negative. In a previous study, 14% of patients with a convincing history of HVA with both negative skin tests and nondetectable venom sIgE subsequently suffered anaphylaxis following Hymenoptera sting challenge [61]. Initial findings suggested molecular allergy diagnostics could improve the diagnostic sensitivity in the detection of HVA in patients testing negative for HBV- and YJV-sIgE [36]. A subsequent study failed to verify this and found no diagnostic benefit of molecular allergy diagnostics in patients with negative skin tests and lack of venom sIgE [42].

### Cellular tests

The basophil activation test (BAT) is not a first-line test but its role in the diagnostics of HVA is well established. It requires expertise with respect to both its practical implementation and interpretation of results and is usually reserved for use in secondary care centers. CD203c and CD63 molecules are both expressed on basophil granule membranes. Following allergen-induced activation, basophils express these molecules on the cell surface and can be quantified by flow cytometry. The sensitivity for the BAT measuring CD63 expression is reported as 89%, for CD203c expression 97% [62]. The use of the CD63 BAT is more widespread. Negative controls in basophil activation tests show a background basophil activation of approx. 10%. As a result a level of 15% basophil activation has been chosen as the cut-off level to identify Hymenoptera venom sensitizations [63].

In patients with no detectable venom sIgE but a convincing history of HVA, an IgE-mediated sensitization can be detected with the BAT in 80% [64] and in 60% of those also negative in skin tests, making it a particularly useful diagnostic tool in this subgroup [65]. Similarly where diagnostics and history show contradictory results, the BAT may detect missed Hymenoptera venom sensitizations. In a study of 63 patients with mastocytosis and a history of HVA but no evidence of sensitization to Hymenoptera venom with sIgE or skin testing, the BAT did not detect any further sensitization [66]. This suggests that the efficacy of the BAT may be reduced in mastocytosis patients or possibly in those with low total IgE levels.

As with skin tests and venom sIgE, the ability of the BAT to provide reliable results is hampered by the presence of CCD in whole venom extracts [67]. BAT has been suggested to be helpful in the investigation of double sensitized patients who reacted to only one sting in the past or in those where molecular-based allergy diagnostics are ambiguous. In particular the BAT using CCD-free species-specific allergens (Ves v 1 and Ves v 5) was shown to improve diagnostic precision in the detection of YJV allergy [63]; however, it is unclear if, in the case of sensitization to cross-reactive allergens such as Ves v 2 and Api m 2 or Ves v 3 and Api m 5, any differentiation between primary and cross-reactive sensitizations is possible. As the BAT is not fully standardized, the results of different studies are difficult to compare. False-positive BAT results may be caused by high venom concentrations. False-negative results may occur with the absolute number of basophils evaluated are less than 150, or as with other diagnostic tests, a long interval between sting event and diagnostic work-up.

In the histamine release test, a precursor of the BAT, histamine released by activated basophils was quantified. The finding that not only basophils but also platelets contributed to histamine release reduced the diagnostic reliability of this test. The histamine release test is laborious, expensive, and has largely been replaced by the BAT. The cellular antigen stimulation test (CAST) measures sulfidoleukotriene release by activated basophils and may be helpful in isolated cases.

### Further diagnostic tests

In some countries, an intentional sting challenge is included in the diagnostic work-up of patients with suspected HVA. If a systemic reaction occurs, an intentional sting challenge confirms the clinical relevance of a sensitization; however, it can lead to severe systemic reactions. The diagnostic sting challenge is therefore highly controversial [68]. It has been argued that a diagnostic sting challenge reduces the socio-economic burden of HVA. Using a diagnostic sting challenge to confirm the clinical relevance of a sensitization, one study group argued that VIT could be withheld from 83% of YJV and 56% of HBV allergic patients, due to tolerance of the diagnostic sting challenge [69]. Another study from the same time showed the diagnostic sting challenge to be unreliable as 21% of patients tolerating an initial sting challenge developed anaphylaxis following a second sting challenge [70]. Importantly, half of those reacting to the second challenge suffered severe anaphylaxis. In Germany, diagnostic sting challenges in the case of HVA are no longer recommended as the risks clearly outweigh the benefits [5].

IgE-inhibition tests with whole venom are expensive and time consuming. Due to the complexity of individual patient sensitization profiles, the added benefit of inhibition tests is probably minimal in most cases. In addition, their use in patients with low sIgE is limited. IgE-inhibition tests may be useful in isolated cases, e. g., for detecting primary sensitizations in patients double sensitized to *Polistes *and YJV, where discriminating marker allergens are not yet available [71].

## Diagnostic algorithm

**Fig. 2:**
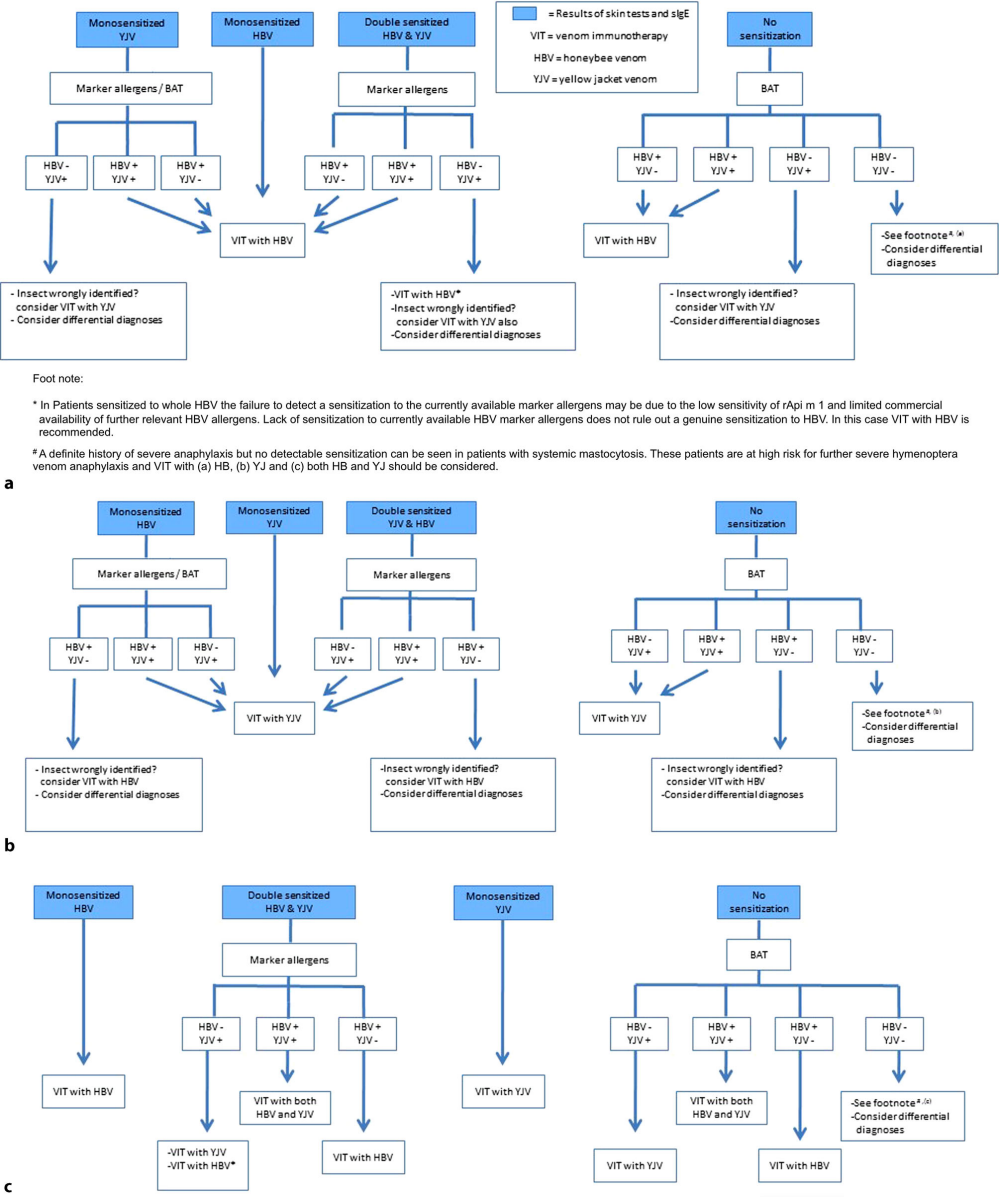
Recommended diagnostic algorithm for the investigation of Hymenoptera venom allergic patients. **a** Insect honeybee (as reported by the patient), **b** Insect yellow jacket (as reported by the patient), and **c** Insect not identified by the patient

All patients with a history of HVA require a basic diagnostic work-up encompassing a medical history, clinical examination, skin testing, and detection of total and venom sIgE to HBV and YJV. For risk stratification, it is useful to determine baseline serum tryptase. The following management algorithm guides the clinician through the steps required to make a competent diagnosis of HVA. The algorithm assists the choice of venom for VIT based on the test results and patient history. The algorithm is summarized in Fig. 2 and assumes differential diagnoses in Table 1 are considered unlikely.

### Culprit insect honeybee according to the patient

Patients reporting honeybee as the culprit insect that are monosensitized to HBV require no further diagnostics and receive honeybee VIT (Fig. 2a). Patients with the same history but monosensitized to YJV should undergo further investigation with HBV marker allergens/BAT. If a HBV sensitization is detected, honeybee VIT is indicated. If a genuine monosensitization to YJV is the only finding, the reason may be incorrect identification of the culprit insect. VIT with YJV should be considered.

Patients double sensitized on basic diagnostic tests, need further investigation with marker allergens in order to clarify the cause of double sensitization. Those with a genuine sensitization to HBV should receive VIT with HBV. Sensitization to YJV can be considered irrelevant. Where only a genuine sensitization to YJV marker allergens is detected, the possibility that the culprit insect was in fact a yellow jacket must be considered. The low sensitivity of rApi m 1 and limited availability of further relevant HBV allergens means a relevant genuine sensitization to HBV cannot be ruled out. VIT with HVB is recommended and VIT with YJV should be considered.

In patients with a definite history of sting-related anaphylaxis but negative diagnostics, VIT with HBV may still be considered, in particular in patients at high risk for severe sting-related anaphylaxis, e. g., mastocytosis patients or those having suffered anaphylaxis with cardiorespiratory arrest. In patients with mast cell disease, the history alone may be the only indication of HVA due to very low levels of circulating IgE.

### Culprit insect yellow jacket according to the patient

Patients reporting yellow jacket as the culprit insect that are monosensitized to YJV receive VIT with YJV (Fig. 2b). Those reporting a yellow jacket but monosensitized to honeybee require further diagnostics with marker allergens/BAT. If a genuine sensitization to YJV is detected, then VIT with YJV is indicated. If diagnostics with marker allergens/BAT contradict the history and detect only a genuine HBV sensitization, the insect may have been wrongly identified and VIT with HBV should be considered.

Patients double sensitized on routine diagnostics require further investigation with marker allergens. Detection of a genuine sensitization to yellow jacket or genuine double sensitization provides a rational for prescribing VIT with YJV. Again if a genuine sensitization to HBV marker allergens is the only finding, then the option of VIT with HBV should be discussed with the patient.

Patients with the same history but no evidence of any sensitization should be further investigated with a BAT. Those sensitized to YJV in the BAT receive VIT with YJV. A HBV sensitization alone suggests the insect was incorrectly identified and VIT with HBV should be considered. In patients with a definite history of severe sting-related anaphylaxis but entirely negative diagnostics, VIT with YJV may be considered in patients at high risk for severe sting-related anaphylaxis.

### Culprit insect not identified by the patient

Patients that were unable to identify the culprit insect and that are monosensitized to HBV on routine diagnostic work-up should receive VIT with HBV (Fig. 2c).

Those double sensitized at this level require testing with marker allergens. Those with evidence only of a genuine sensitization to HBV marker allergens require VIT with HBV; those genuinely double sensitized receive double VIT. In those only genuinely sensitized to YJV marker allergens, the relatively low sensitivity of Api m 1 and Api m 10, and limited availability of further marker allergens means VIT with YJV is indicated and additional VIT with HBV should still be considered.

Patients that are monosensitized to YJV on routine diagnostics receive VIT with YJV. When basic diagnostics reveal no sensitizations, a BAT should be performed and VIT chosen according to the sensitization profile obtained. If no sensitizations are detected with this step, but there is a definite history of severe sting-related anaphylaxis, VIT with HVB and YJV may be considered in patients at high risk for severe sting-related anaphylaxis.

## Perspective

Optimal management of HVA patients can be challenging. Molecular allergy diagnostics have significantly improved the diagnostic precision in HVA but a diagnostic gap remains. Until recently, the main limiting factor has been the commercial availability of only a few marker allergens (Ves v 1, Ves v 5, Api m 1, and Api m 10). The release of Api m 2, Api m 3, and Api m 5 as additional HBV allergens in 2016 will further improve diagnostic accuracy in the future. Our own data showed that the combination of Api m 1, 2, 3, 4, 5, and 10 detected 94% of honeybee venom allergic patients. We speculate that the commercial availability of further, albeit cross-reactive HBV allergens may help to further differentiate primary honeybee and yellow jacket sensitizations. The homology between cross-reactive allergens of HBV and YJV reaches 45–50%. We hypothesize that comparing the magnitude of sensitizations to cross-reactive homologous allergens, e. g., Ves v 2 and Api m 2 or Ves v 3 and Api m 5, may help to identify primary sensitizations, as a greater degree of sensitization, i. e., IgE reactivity, would be expected to the clinically relevant venom. Similarly it remains to be seen what role the BAT with CCD-free cross-reactive allergens may have in improving the diagnostic sensitivity in patients double sensitized to HBV and YJV. Finally, molecular sensitization profiles may not only help us to improve diagnostic precision, but may also prove to be useful as risk markers for treatment failure in VIT, as quite recently demonstrated for dominant Api m 10 sensitization in HBV allergy [72].

## Abbreviations

BAT: Basophil activation test
CAST: Cellular antigen stimulation test
CCD: Cross-reactive carbohydrate determinant
HBV: Honeybee venom
HRP: Horseradish peroxidase
HVA: Hymenoptera venom allergy
sIgE: Specific IgE antibodies
SPT: Skin prick test
VIT: Venom immunotherapy
YJV: Yellow jacket venom
